# Loss of imprinting at the *Dlk1*-*Gtl2 *locus caused by insertional mutagenesis in the *Gtl2 *5' region

**DOI:** 10.1186/1471-2156-7-44

**Published:** 2006-10-03

**Authors:** Ekaterina Y Steshina, Michael S Carr, Elena A Glick, Aleksey Yevtodiyenko, Oliver K Appelbe, Jennifer V Schmidt

**Affiliations:** 1 Department of Biological Sciences, The University of Illinois at Chicago, 900 S. Ashland Avenue, MC 567, Chicago, IL 60607, USA

## Abstract

**Background:**

The *Dlk1 *and *Gtl2 *genes define a region of mouse chromosome 12 that is subject to genomic imprinting, the parental allele-specific expression of a gene. Although imprinted genes play important roles in growth and development, the mechanisms by which imprinting is established and maintained are poorly understood. Differentially methylated regions (DMRs), which carry methylation on only one parental allele, are involved in imprinting control at many loci. The *Dlk1*-*Gtl2 *region contains three known DMRs, the *Dlk1 *DMR in the 3' region of *Dlk1*, the intergenic DMR 15 kb upstream of *Gtl2*, and the *Gtl2 *DMR at the *Gtl2 *promoter. Three mouse models are analyzed here that provide new information about the regulation of *Dlk1*-*Gtl2 *imprinting.

**Results:**

A previously existing insertional mutation (*Gtl2lacZ*), and a targeted deletion in which the *Gtl2 *upstream region was replaced by a *Neo *cassette (*Gtl2Δ5'Neo*), display partial lethality and dwarfism upon paternal inheritance. Molecular characterization shows that both mutations cause loss of imprinting and changes in expression of the *Dlk1*, *Gtl2 *and *Meg8*/*Rian *genes. *Dlk1 *levels are decreased upon paternal inheritance of either mutation, suggesting *Dlk1 *may be causative for the lethality and dwarfism. Loss of imprinting on the paternal chromosome in both *Gtl2lacZ *and *Gtl2Δ5'Neo *mice is accompanied by the loss of paternal-specific *Gtl2 *DMR methylation, while maternal loss of imprinting suggests a previously unknown regulatory role for the maternal *Gtl2 *DMR. Unexpectedly, when the *Neo *gene is excised, *Gtl2Δ5' *animals are of normal size, imprinting is unchanged and the *Gtl2 *DMR is properly methylated. The exogenous DNA sequences integrated upstream of *Gtl2 *are therefore responsible for the growth and imprinting effects.

**Conclusion:**

These data provide further evidence for the coregulation of the imprinted *Dlk1 *and *Gtl2 *genes, and support a role for *Dlk1 *as an important neonatal growth factor. The ability of the *Gtl2lacZ *and *Gtl2Δ5'Neo *mutations to cause long-range changes in imprinting and gene expression suggest that regional imprinting regulatory elements may lie in proximity to the integration site.

## Background

**Figure 1:**
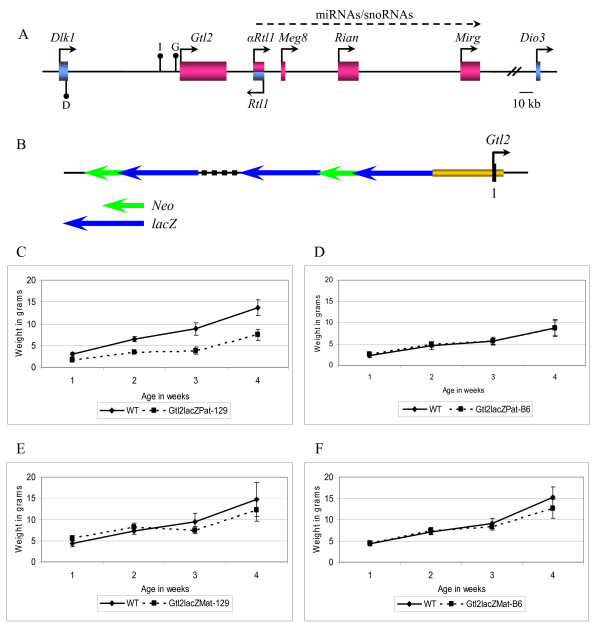
***Dlk1*-*Gtl2 *domain and *Gtl2lacZ *integration**. A) Schematic of the *Dlk1*-*Gtl2 *imprinted domain. Maternally expressed genes are shown in pink and paternally expressed genes in blue; the direction of their transcription is indicated. Black lollipops indicate the positions of the three known *Dlk1*-*Gtl2 *DMRs, which have been designated with individual letters, D, *Dlk1 *DMR, I, IG DMR, and G, *Gtl2 *DMR. The dotted arrow indicates a genomic region that produces multiple miRNAs and snoRNAs; emerging evidence suggests contiguous transcription through this region. B) Diagram of the *Gtl2lacZ *integration. The blue arrows represent the *lacZ *genes and the green arrows represent the *Neo *genes. The broken line represents an internal rearrangement that has been refractory to analysis; its size is estimated by Southern blotting. The gold box represents the *Gtl2 *DMR. C) Growth curves of *Gtl2lacZPat*^129 ^mice in comparison to wild type littermates (WT, n = 11, *Gtl2lacZPat*^129^, n = 3). D) Growth curves of *Gtl2lacZPat*^*B*6 ^mice in comparison to wild type littermates (WT, n = 6, *Gtl2lacZPat*^*B*6^, n = 7). E) Growth curves of *Gtl2lacZMat*^129 ^mice in comparison to wild type littermates (WT, n = 7, *Gtl2lacZMat*^129^, n = 8). F) Growth curves of *Gtl2lacZMat*^*B*6 ^mice in comparison to wild type littermates (WT, n = 7, *Gtl2lacZMat*^*B*6^, n = 8). In all graphs the solid line represents wild type animals and the dashed line represents *Gtl2lacZ *animals.

The paternally expressed *Delta-like 1 *(*Dlk1*) and maternally expressed *Gene-trap locus 2 *(*Gtl2*) genes lie within an imprinted gene cluster on distal mouse chromosome 12 [1-5]. This gene cluster spans 1 Mb of DNA, and contains several linked imprinted genes (Fig. 1A). In addition to *Dlk1 *and *Gtl2*, these include the paternally expressed *Dio3 *and *Peg11*/*Rtl1 *genes, and the maternally expressed *αPeg11*/*αRtl1*, *Meg8*/*Rian *and *Mirg *genes [6-13]. The *Dlk1 *gene produces a Notch family transmembrane protein, and the *Dio3 *gene produces an enzyme involved in thyroid hormone metabolism. *Peg11*/*Rtl1 *is a retrotransposon-like gene, *αPeg11*/*αRtl1 *and *Mirg *produce multiple miRNAs and the *Meg8*/*Rian *gene produces a series of C/D snoRNAs [10,11,14,15]. Such clustering of imprinted genes is common, and is believed to indicate coordinate regulation of multiple genes.

Many imprinted genes function as regulators of mammalian development, particularly in the control of cell growth and differentiation. These roles are illustrated by loss of imprinting mutations in humans, which frequently result in growth disorders or cancer [16]. There is evidence that *Dlk1 *is a growth regulator, and the *Dlk1 *transcript is overexpressed in many human neuroendocrine tumors [17-19]. DLK1 protein has been shown to prevent differentiation of preadipocytes, and to promote the expansion of undifferentiated cell populations in the bone marrow and thymus [20-24]. Mice carrying a targeted mutation of the *Dlk1 *gene show partial neonatal lethality, and surviving animals are growth retarded and display skeletal and adipose phenotypes [25]. The *Gtl2 *gene produces a noncoding RNA product of unknown function that is present in many different splice forms. *Gtl2 *was identified by an insertional mouse mutation, *Gtl2lacZ*, in which multiple copies of a *lacZ*-*Neo *gene trap vector integrated upstream of *Gtl2 *(Fig. 1B) [26]. Paternal, but not maternal, inheritance of this mutation causes reduced survival and a proportional dwarfism in surviving animals [27]. This phenotype suggests the integration altered the expression and/or imprinting of one or more genes involved in growth regulation, but the *Gtl2lacZ *mutation has not been characterized at the molecular level.

Imprinted genes are commonly accompanied by regions of CpG-rich sequence that carry allele-specific methylation, the methylation of one parental allele but not the other. These differentially methylated regions (DMRs) often contain imprinting regulatory elements for one or more genes. DNA methylation plays an important role in *Dlk1*-*Gtl2 *regulation; in DNA methyltransferase deficient (*Dnmt1*^-/-^) mice the paternal *Dlk1 *allele is repressed and the silent allele of *Gtl2 *is activated [1]. At the *Dlk1*-*Gtl2 *locus, three DMRs have been defined, the *Dlk1 *DMR, in the 3' region of the *Dlk1 *gene, the intergenic DMR (IG DMR) located 15 kb upstream of *Gtl2*, and the *Gtl2 *DMR located across the *Gtl2 *promoter (Fig. 1A) [1,28]. These regions are methylated on the paternally inherited allele, although only the IG DMR acquires this methylation in the germline. This suggests the IG DMR may represent the gametic mark for the *Dlk1*-*Gtl2 *genes, but it alone cannot explain imprinting within the region. Mice inheriting an IG-DMR deletion maternally show a maternal to paternal epigenotype switch, with overexpression of paternally expressed genes and silencing of maternally expressed genes [29]. This deletion has no effect when inherited paternally, however, so the mechanisms regulating *Dlk1*-*Gtl2 *imprinting on the paternal chromosome are unknown.

This paper describes the analysis of three mouse models that suggest the *Gtl2 *DMR may play a role in regulating the imprinting of multiple genes within the *Dlk1*-*Gtl2 *domain. The previously generated *Gtl2lacZ *mutation is shown to alter the expression and imprinting of *Dlk1 *and *Gtl2 *on both parental chromosomes. After either paternal or maternal inheritance, the gene(s) that should be silent on the mutant chromosome are activated, and the normally active gene(s) are repressed. To better understand the role of the region disrupted by the *Gtl2lacZ *integration, a deletion of the integration site was generated by gene targeting. Targeted mice in which the Neomycin resistance gene replaces the *Gtl2 *upstream region (*Gtl2Δ5'Neo *allele) phenocopy both the physiological (neonatal lethality and dwarfism) and molecular (loss of imprinting) effects of the *Gtl2lacZ *integration. Upon paternal inheritance, both the *Gtl2lacZ *and *Gtl2Δ5'Neo *mutations cause a loss of *Gtl2 *DMR methylation on the paternal allele, suggesting this methylation may be involved in maintaining imprinting on the paternal chromosome. The reciprocal loss of imprinting after maternal inheritance also implies a role for the unmethylated maternal *Gtl2 *upstream region in regulating imprinting on that chromosome. When the *Neo *gene is removed by Cre-mediated excision (*Gtl2Δ5' *allele), however, the animals are of normal size, proper imprinting is restored and the allele-specific methylation of the *Gtl2 *DMR is recovered. The data presented here suggest that 1) the imprinting of genes in the *Dlk1*-*Gtl2 *locus is coordinately regulated, 2) this regulation may require the allele-specific methylation of the *Gtl2 *DMR, 3) the presence of exogenous DNA sequences adjacent to the *Gtl2 *DMR prevents this methylation, and 4) decreased levels of *Dlk1 *may be causative for the lethality and dwarfism of the *Gtl2lacZ *and *Gtl2Δ5'Neo *paternal mutants.

## Results

### *Gtl2lacZ *mice display a paternally inherited dwarfism

The *Gtl2lacZ *mouse strain provided a model system to investigate the function and regulation of imprinted genes at the *Dlk1*-*Gtl2 *locus. These mice carry a 15-kb integration with 2–3 copies of a promoterless-*lacZ*/*β-actin-Neo *transgene upstream of the *Gtl2 *DMR (Fig. 1B) [26,30]. The *Gtl2lacZ *mice were generated on a mixed C57BL/6 (B6) and 129/Sv (129) background. Paternal inheritance of this integration was shown previously to confer reduced survival, and proportional dwarfism of surviving animals, on a 129 background, but a less severe phenotype with no lethality on a mixed (B6 × BALB/c) background [26]. The parental-specific inheritance of the *Gtl2lacZ *phenotype suggested that this mutation affected the function or regulation of imprinted gene(s) near the integration.

**Figure 9:**
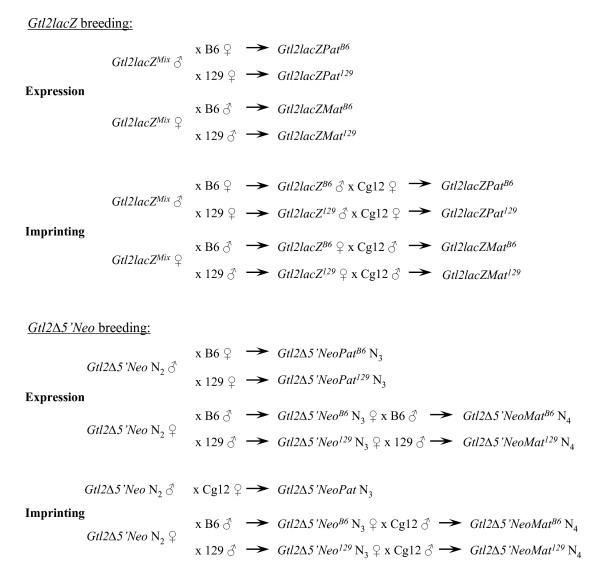
**Breeding strategies for the *Gtl2lacZ *and *Gtl2Δ5'Neo *mice**. The crosses generated for gene expression and imprinting analysis are detailed. In each cross, the final animals examined for expression/imprinting are indicated at the far right.

*Gtl2lacZ *mice were obtained on a mixed genetic background, and initial analysis of the animals confirmed the previously reported dwarfism phenotype and the presence of strain-specific modifiers. The offspring of a mixed background *Gtl2lacZ *male were dwarf when the mother was a 129 female, but were of normal size when the mother was a B6 female (data not shown). In an attempt to generate pure 129 strain *Gtl2lacZ *mice for analysis, the transgene was introgressed into the 129 background. A separate group of animals was maintained on a mixed B6/129 background by alternating strains at each generation. After only 3–4 backcrosses onto the 129 background, the dwarfism phenotype diminished and then disappeared. Multiple outcrosses to B6 were performed in an attempt to recover the dwarfism, but this was not successful. The *Gtl2lacZ *animals analyzed in this work are therefore those that were maintained on a mixed background. For analysis of the growth phenotype and gene expression after paternal inheritance, a mixed background male was mated to a 129 or B6 female and the offspring analyzed. For maternal inheritance, a mixed background female was mated to a 129 or B6 male and the offspring analyzed (see Fig. 9 for a detailed breeding schematic). The direction of inheritance of the transgene and the strain of the wild type animal are indicated in the names of the offspring, with "Pat" or "Mat" indicating paternal or maternal inheritance, and the superscripts 129 or B6 indicating the strain of the wild type partner.

Analysis of the *Gtl2lacZ *animals began with characterization of survival and growth after paternal and maternal inheritance of the transgene. Heterozygous animals inheriting the integration paternally (*Gtl2lacZPat*) and maternally (*Gtl2lacZMat*) were weighed weekly from 1 to 4 weeks of age. Similar to previously published data [26], *Gtl2lacZPat*^129 ^animals showed neonatal lethality, and survivors were smaller than their wild-type littermates at all time points analyzed, weighing 54% of wild type at 4 weeks of age (Fig. 1C). Of 21 individual *Gtl2lacZPat*^129 ^animals followed, six mice died between one and two weeks of age, a lethality rate of 29% (data not shown). None of the wild type littermates died in this experiment. *Gtl2lacZPat*^*B*6 ^mice, however, showed no lethality or weight difference from their wild type littermates (Fig. 1D). For unknown reasons, wild type littermates were also smaller in this cross. Larger litter sizes can result in an overall reduction in pup weight, but the average litter sizes were similar for *Gtl2lacZPat*^129 ^and *Gtl2lacZPat*^*B*6 ^crosses (5.4 and 6.0 pups, respectively). Genetic background therefore has a significant modifying effect on the severity of the *Gtl2lacZPat *phenotype. *Gtl2lacZMat*^129 ^and *Gtl2lacZMat*^*B*6 ^mice showed no differences in weight from their wild type littermates (Fig. 1E,F).

### Characterization of the *Gtl2lacZ *integration

The dwarfism phenotype of *Gtl2lacZPat*^129 ^animals suggested that the integration altered the expression of a gene involved in growth. Since the integration site is intergenic, it was likely that a cis-acting regulatory element for a nearby gene was disrupted. The position of the *Gtl2lacZ *transgene had been reported to lie 3 kb upstream of the *Gtl2 *promoter [27], but attempts to confirm this position were unsuccessful, suggesting it might be incorrect. A combination of PCR and sequencing was therefore used to map the *Gtl2lacZ *integration site. Genomic DNA from *Gtl2lacZ *mice was amplified with the Gtl2lacZ4 and Gtl2trlacZ primers, and the resulting PCR product spans the distal boundary of the transgene insertion. Sequencing of the fragment localized the integration to a position 1659 bp upstream of the *Gtl2 *transcriptional initiation site (data not shown). As these experiments were in progress, another group also localized the *Gtl2lacZ *integration to this position [31]. Integration of foreign DNA into the mouse genome is often imprecise, with deletion of endogenous sequences at the integration site. Attempts to identify any deletion upstream of the transgene array were not successful, likely because the repetitive nature of the array complicates PCR analysis. Detailed Southern mapping carried out in the initial *Gtl2lacZ *report, however, did not detect a deletion [27].

### *Gtl2lacZ *mice show altered expression and imprinting of *Dlk1-Gtl2*

**Figure 2:**
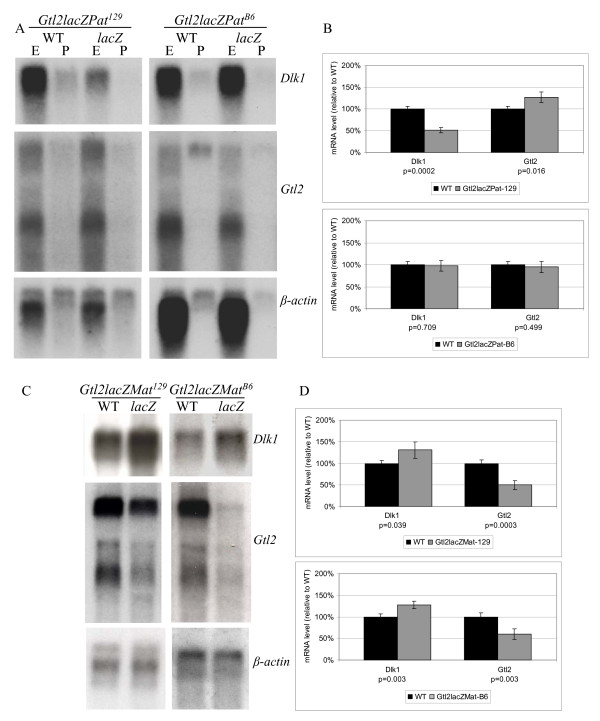
**Expression levels of *Dlk1 *and *Gtl2 *mRNAs in *Gtl2lacZ *embryos**. A) Representative Northern blots for *Dlk1 *and *Gtl2 *RNA levels, in comparison to β*actin*, in G*tl2lacZPat*^129 ^(left) and *Gtl2lacZPat*^*B*6 ^(right) embryo and placenta. B) Graphical representation of quantitative expression data for *Gtl2lacZPat*^129 ^(top) and *Gtl2lacZPat*^*B*6 ^(bottom) embryos. In each graph, the black bars represent wild type (the mean of these values was set to 100), and the gray bars represent *Gtl2lacZPat*, E indicates embryo samples and P indicates placental samples; quantitative data is given for embryo only. P-values are indicated below each graph. (Top graph, *Dlk1*, WT, n = 3, *Gtl2lacZPat*^129^, n = 4; *Gtl2*, WT, n = 3, *Gtl2lacZPat*^129^, n = 4. Bottom graph, *Dlk1*, WT, n = 4, *Gtl2lacZPat*^*B*6^, n = 4; *Gtl2*, WT, n = 4, *Gtl2lacZPat*^*B*6^, n = 4). C) Representative Northern blots for *Dlk1 *and *Gtl2 *RNA levels, in comparison to β-*actin*, in *Gtl2lacZMat*^129 ^(left) and *Gtl2lacZMat*^*B*6 ^(right) embryos. D) Graphical representation of quantitative expression data for *Gtl2lacZMat*^129 ^(top) and *Gtl2lacZMat*^*B*6 ^(bottom) embryos. In each graph, the black bars represent wild type (the mean of these values was set to 100), and the gray bars represent *Gtl2lacZMat*. P-values are indicated below each graph. Differences in β-actin levels are the result of analyzing different midgestation embryonic stages. (Top graph, *Dlk1*, WT, n = 4, *Gtl2lacZMat*^129^, n = 4; *Gtl2*, WT, n = 4, *Gtl2lacZMat*^129^, n = 4. Bottom graph, *Dlk1*, WT, n = 4, *Gtl2lacZMat*^*B*6^, n = 4; *Gtl2*, WT, n = 4, *Gtl2lacZMat*^*B*6^, n = 4). All expression data includes embryos from at least two litters.

To understand the molecular basis of the *Gtl2lacZ *phenotype, levels of *Dlk1 *and *Gtl2 *mRNAs were assayed by Northern blotting in midgestation *Gtl2lacZPat *and *Gtl2lacZMat *embryos. The *Dlk1 *gene produces a single 1.6 kb transcript by Northern analysis, but the noncoding RNA produced from the *Gtl2 *gene is alternatively and only partially spliced, resulting in a smear of expression with a few more prominent splice forms. The pattern of these forms varies with developmental stage, but is always similar between littermates. *Gtl2 *expression was therefore quantified using the intensity of the entire smear, a method that has proven reliable during several studies of *Gtl2 *expression [1,29,32]. Northern blotting of RNA from *Gtl2lacZPat*^129 ^embryos showed that *Dlk1 *expression was 52% of the level found in wild type littermates, and *Gtl2 *expression was 127% of wild type (Fig. 2A,B). No changes in gene expression were observed in *Gtl2lacZPat*^*B*6 ^embryos, consistent with the lack of dwarfism in these animals (Fig. 2A,B). *Dlk1*-*Gtl2 *levels were also analyzed in the placenta, and showed equivalent changes to those seen in embryo (Fig. 2A). The levels of *Gtl2 *are low in placenta, however, and quantitation proved difficult, so placental expression was not examined further. *Gtl2lacZMat *embryos showed changes in gene expression reciprocal to those seen in *Gtl2lacZPat*, despite the lack of any observable growth phenotype. *Gtl2lacZMat*^129 ^embryos had *Dlk1 *levels that were 131% of wild type, and *Gtl2 *levels 50% of wild type, while *Gtl2lacZMat*^*B*6 ^embryos displayed *Dlk1 *levels 127% of wild type and *Gtl2 *levels 60% of wild type (Fig. 2C,D) (Table 1). The *Gtl2lacZ *integration therefore affects the expression of *Dlk1*-*Gtl2 *after paternal inheritance only when crossed to 129 mice, but after maternal inheritance when crossed to either 129 or B6 animals.

**Table 1:** Summary of *Dlk1-Gtl2 *expression and imprinting in *Gtl2lacZ *and *Gtl2Δ5'Neo *mice.

	***Dlk1***		***Gtl2***	
	**Expression**	**Imprinting**	**Expression**	**Imprinting**

*Gtl2lacZPat*^129^	52%	No change	127%	Biallelic
*Gtl2lacZPat*^*B*6^	No change	No change	No change	No change
*Gtl2lacZMat*^129^	131%	Biallelic	50%	No change
*Gtl2lacZMat*^*B*6^	127%	Biallelic	60%	No change
				
	***Dlk1***		***Gtl2***	

	**Expression**	**Imprinting**	**Expression**	**Imprinting**

*Gtl2Δ5'NeoPat*^129^	60%	No change	132%	Biallelic
*Gtl2Δ5'NeoPat*^*B*6^	35%	No change	160%	Biallelic
*Gtl2Δ5'NeoMat*^129^	108%	Biallelic	83%	No change
*Gtl2Δ5'NeoMat*^*B*6^	127%	Biallelic	55%	No change

The column "Expression" gives percent change in mRNA levels relative to wild type littermates; "No change" indicates that there is no deviation in expression from wild type. The column "Imprinting" indicates that imprinting is maintained ("No change") or that the silent allele of a given gene is activated ("biallelic").

Increased expression of an imprinted gene results either from increased transcription of the normally active allele of the gene, or from activation of the normally silent allele. Imprinting assays have been described previously for the *Dlk1 *and *Gtl2 *genes, based on polymorphisms between the *Mus musculus domesticus *and *Mus musculus castaneus *mouse subspecies [1]. Also previously reported is the congenic mouse line B6.CAST-D12Mit30-D12Mit263 (abbreviated Cg12), which carries a *M. m. castaneus*-derived distal chromosome 12 on a B6 background [6]. Embryos were analyzed at midgestation for the imprinting status of *Dlk1 *and *Gtl2*; in these crosses, the *M. m. domesticus *allele of the *Gtl2lacZ *animal is abbreviated as "D", and the *M. m. castaneus *allele of the Cg12 animal is abbreviated as "C". Certain interspecific mouse crosses have been reported to show loss of imprinting in wild type animals [33-35]. In contrast to these systems, however, the Cg12 mice carry only distal chromosome 12 from Cast/Ei, and have been used to analyze over one hundred wild type animals with no loss of imprinting observed [6,32] (JVS, unpublished data).

**Figure 3:**
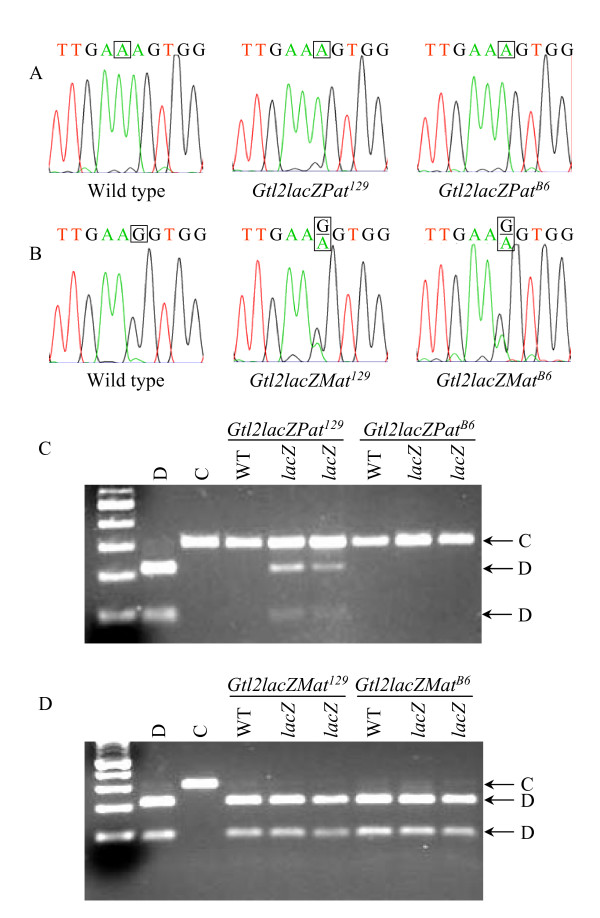
***Dlk1*-*Gtl2 *imprinting analysis in *Gtl2lacZ *embryos**. A) Imprinting assay for *Dlk1 *in wild type (left), *Gtl2lacZPat*^129 ^(center) and *Gtl2lacZPat*^*B*6 ^(right) embryos. B) Imprinting assay for *Dlk1 *in wild type (left), *Gtl2lacZMat*^129 ^(center) and *Gtl2lacZMat*^*B*6 ^(right) embryos. The *Dlk1 *polymorphism is an "A" in D embryos and a "G" in C embryos; the polymorphic base is boxed on each chromatogram. C) Imprinting assay for *Gtl2 *in *Gtl2lacZPat*^129 ^and *Gtl2lacZPat*^*B*6 ^embryos. D) Imprinting assay for *Gtl2 *in *Gtl2lacZMat*^129 ^and *Gtl2lacZMat*^*B*6 ^embryos. The *Gtl2 *polymorphism alters an *Sfc*I restriction enzyme site, with the site present in D and absent in C; the D and C bands are indicated at the right.

*Gtl2lacZ *transgenic animals were crossed to Cg12 mice to assay for imprinting, using a breeding scheme as follows (see Fig. 9). For paternal inheritance, a mixed background male was crossed to a 129 or B6 female, then the male pups from this cross were bred to a Cg12 female and midgestation embryos collected for analysis. To analyze maternal inheritance, a mixed background female was crossed to a 129 or B6 male, then the female pups from this cross bred to a Cg12 male and embryos collected for analysis. The *Dlk1 *imprinting assay involves RT-PCR amplification of the *Dlk1 *cDNA, followed by direct sequencing to detect an A to G polymorphism, where the D strain carries an "A" and the C strain carries a "G". In crosses between a Cg12 female and either a *Gtl2lacZ*^129 ^or *Gtl2lacZ*^*B*6 ^male, the resulting *Gtl2lacZPat*^129 ^and *Gtl2lacZPat*^*B*6 ^embryos showed the presence of only D RNA, indicating exclusive expression of the paternal *Dlk1 *allele (Fig. 3A). *Dlk1 *imprinting is therefore retained in *Gtl2lacZPat *embryos. In crosses to analyze maternal inheritance, *Gtl2lacZMat*^129 ^and *Gtl2lacZMat*^*B*6 ^embryos expressed both the D and C *Dlk1 *alleles (Fig. 3B). The increased level of *Dlk1 *found in these animals therefore results from loss of imprinting, with activation of the normally silent maternal *Dlk1 *allele.

The *Gtl2 *imprinting assay employs a polymorphism between D and C mice that alters an *Sfc*I restriction site, with the site present in D mice and absent in C mice, so RT-PCR followed by restriction digestion was used to analyze *Gtl2 *imprinting. *Gtl2lacZPat*^129 ^embryos showed expression of both the D and C alleles of *Gtl2 *(Fig. 3C), indicating that the increased level of *Gtl2 *in these animals results from loss of imprinting, with activation of the normally silent paternal *Gtl2 *allele. *Gtl2lacZPat*^*B*6 ^embryos, however, showed no change in *Gtl2 *imprinting, consistent with the lack of any change in gene expression in these animals. *Gtl2lacZMat*^129 ^and *Gtl2lacZMat*^*B*6 ^embryos expressed only the D allele, indicating that *Gtl2 *imprinting is maintained in these animals (Fig. 3D). *Gtl2lacZ *mice, therefore, show loss of imprinting of the normally silent gene on the chromosome that carries the integration – *Dlk1 *in *Gtl2lacZMat *animals and *Gtl2 *in *Gtl2lacZPat*^129 ^animals (Table 1). In both cases, the gene that is overexpressed is subject to a loss of imprinting, and the gene that retains imprinting is decreased in expression.

### Generation of a *Gtl2 *upstream deletion

**Figure 4:**
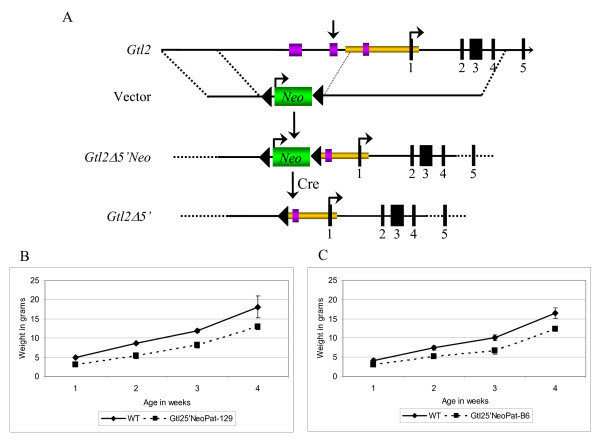
**Gene targeting of the *Gtl2 *upstream region**. A) Schematic representation of the *Gtl2 *upstream region and the *Gtl2Δ5'Neo *and *Gtl2Δ5' *targeted loci. The targeting event replaces a 2.8-kb region of *Gtl2 *upstream sequence with the *Neo *gene (*Gtl2Δ5'Neo *allele). Cre-mediated excision removes the *Neo *gene, leaving a single loxP site in the locus (*Gtl2Δ5' *allele). The black boxes represent the *Gtl2 *exons (only exons 1–5 are shown), the gold box represents the *Gtl2 *DMR, the green box represents the *Neo *gene, the purple boxes represent highly conserved regions across species and the vertical arrow indicates the position of the *Gtl2lacZ *integration. B) Growth curves of *Gtl2Δ5'NeoPat*^129 ^mice in comparison to wild type littermates (WT, n = 4, *Gtl2Δ5'NeoPat*^129^, n = 8). C) Growth curves of *Gtl2Δ5'NeoPat*^*B*6 ^mice in comparison to wild type littermates (WT, n = 11, *Gtl2Δ5'NeoPat*^*B*6^, n = 5). In all graphs the solid line represents wild type animals and the dashed line represents *Gtl2Δ5'Neo *animals.

The phenotype of the *Gtl2lacZ *mice suggested that the *Gtl2lacZ *integration region plays a role in the establishment and/or maintenance of *Dlk1*-*Gtl2 *imprinting, and this function was disrupted by the integration. To better define the sequences involved, a targeted deletion was generated that spans the region from -1.3 kb to -4.1 kb relative to the *Gtl2 *transcriptional initiation site (Fig. 4A). This region was chosen because it contains: 1) two short sequences that are greater than 90% conserved between the mouse, human and sheep genomes, 2) a series of degenerate repeats, sequence elements often found near imprinted genes, and 3) the position of the *Gtl2lacZ *integration. Importantly, this deletion leaves the *Gtl2 *gene, *Gtl2 *promoter and all but 100 bp of the *Gtl2 *DMR intact. This was important to preserve normal *Gtl2 *promoter function, to avoid the confounding effect of a *Gtl2 *deletion. The *Gtl2Δ5'Neo *targeting vector carried 1.4 kb of sequence upstream of the region to be deleted, and 6.0 kb downstream (Fig. 4A). Following homologous recombination, the deleted region is replaced with the Neomycin resistance gene flanked by loxP sites. Two correctly targeted ES cell clones were microinjected into B6 blastocysts, and both generated high percentage chimeric animals. Male chimeras were bred to B6 females, and one line transmitted the *Gtl2Δ5'Neo *allele through the germline.

### *Gtl2Δ5'Neo *mice phenocopy the *Gtl2lacZ *transgene integration

*Gtl2Δ5'Neo *mice were bred using a strategy similar to that of the *Gtl2lacZ *mice (see Fig. 9). To assay the effect of paternal inheritance of the mutation, N_2 _males were crossed to 129 or B6 females and their offspring analyzed for growth and gene expression. To assay maternal inheritance, limiting numbers of *Gtl2Δ5'Neo *females required a strategy in which N_2 _females were crossed to 129 or B6 males, and their female pups crossed again to a male of the same strain (129 or B6). The animals analyzed after paternal inheritance were therefore N_3 _from the chimera, while for maternal inheritance they were N_4_. As observed in *Gtl2lacZ *mice, there were strain-specific differences in expressivity of the mutant phenotype. *Gtl2Δ5'NeoPat*^129 ^mice were fully viable but smaller than their wild type littermates, weighing 72% of wild type at 4 weeks of age (Fig. 4B). In contrast to *Gtl2lacZPat*^*B*6^, however, *Gtl2Δ5'NeoPat*^*B*6 ^mice showed a more severe phenotype, with 50% postnatal lethality by 2 weeks of age. Surviving *Gtl2Δ5'NeoPat*^*B*6 ^mice were 75% the size of their wild type littermates at 4 weeks of age (Fig. 4C). Neither *Gtl2Δ5'NeoMat*^129 ^or *Gtl2Δ5'NeoMat*^*B*6 ^mice showed any growth phenotype (data not shown).

### *Gtl2Δ5'Neo *mice display altered expression and imprinting of *Dlk1-Gtl2*

**Figure 5:**
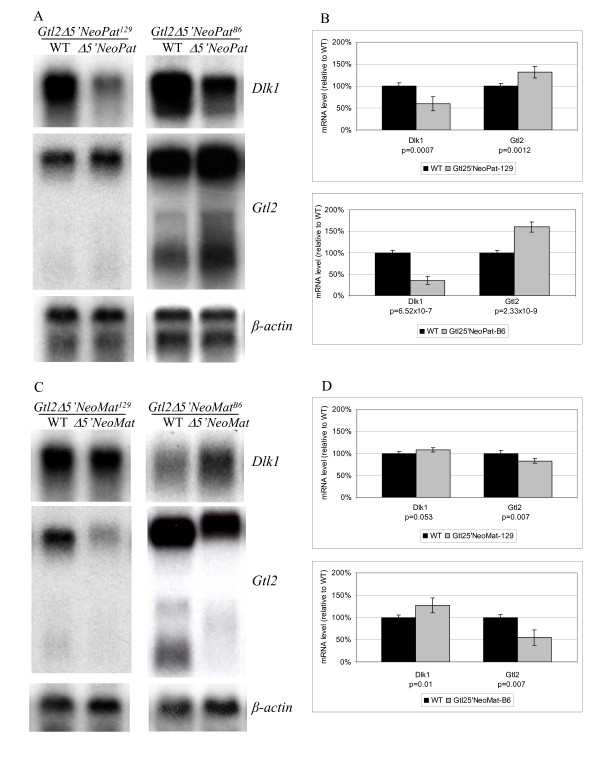
**Expression analysis for *Dlk1*-*Gtl2 *mRNA in *Gtl2Δ5'Neo *embryos**. A) Representative Northern blots for *Dlk1 *and *Gtl2 *RNA levels, in comparison to β-*actin*, in *Gtl2Δ5'NeoPat*^129 ^(left) and *Gtl2Δ5'NeoPat*^*B*6 ^(right) embryos. B) Graphical representation of quantitative expression data for *Gtl2Δ5'NeoPat*^129 ^(top) and *Gtl2Δ5'NeoPat*^*B*6 ^(bottom) embryos. In each graph, the black bars represent wild type (the mean of these values was set to 100), and the gray bars represent *Gtl2Δ5'NeoPat*. P-values are indicated below each graph. (Top graph, *Dlk1*, WT, n = 4, *Gtl2Δ5'NeoPat*^129^, n = 6; *Gtl2*, WT, n = 4, *Gtl2Δ5'NeoPat*^129^, n = 6. Bottom graph, *Dlk1*, WT, n = 6, *Gtl2Δ5'NeoPat*^*B*6^, n = 10; *Gtl2*, WT, n = 6, *Gtl2Δ5'NeoPat*^*B*6^, n = 10). C) Representative Northern blots for *Dlk1 *and *Gtl2 *RNA levels, in comparison to β-*actin*, in *Gtl2Δ5'NeoMat*^129 ^(left) and *Gtl2Δ5'NeoMat*^*B*6 ^(right) embryos. D) Graphical representation of quantitative expression data for *Gtl2Δ5'NeoMat*^129 ^(top) and *Gtl2Δ5'NeoMat*^*B*6 ^(bottom) embryos. In each graph, the black bars represent wild type (the mean of these values was set to 100), and the gray bars represent *Gtl2Δ5'NeoMat*. P-values are indicated below each graph. (Top graph, *Dlk1*, WT, n = 4, *Gtl2Δ5'NeoMat*^129^, n = 6; *Gtl2*, WT, n = 4, *Gtl2Δ5'NeoMat*^129^, n = 6. Bottom graph, *Dlk1*, WT, n = 4, *Gtl2Δ5'NeoMat*^*B*6^, n = 4; *Gtl2*, WT, n = 4, *Gtl2Δ5'NeoMat*^*B*6^, n = 4).

Northern analysis of *Dlk1*-*Gtl2 *expression in *Gtl2Δ5'Neo *embryos showed alterations that were similar, but not identical, to those observed in the *Gtl2lacZ *mice. *Gtl2Δ5'NeoPat*^129 ^embryos had *Dlk1 *levels that were 60% of wild type littermates, and *Gtl2 *levels 132% of wild-type, while *Gtl2Δ5'NeoPat*^*B*6 ^embryos had *Dlk1 *levels 35% of wild type, and *Gtl2 *levels 160% of wild type (Fig. 5A,B). *Gtl2Δ5'NeoMat*^129 ^embryos displayed *Dlk1 *levels 108% of wild type, and *Gtl2 *levels 83% of wild type, while *Gtl2Δ5'NeoMat*^*B*6 ^embryos had *Dlk1 *levels 127% of wild type, and *Gtl2 *levels 55% of wild-type (Fig. 5C,D) (Table 1).

**Figure 6:**
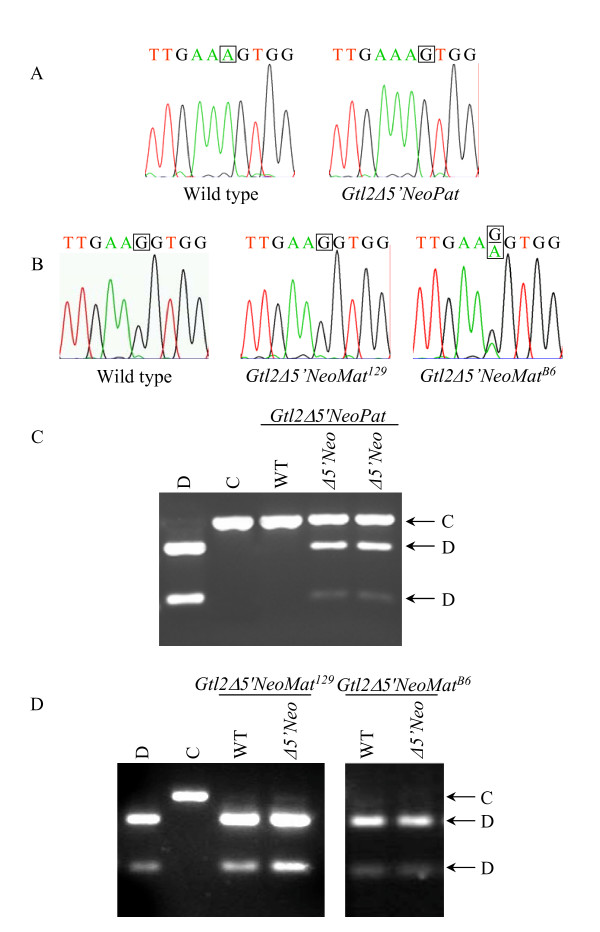
***Dlk1*-*Gtl2 *imprinting analysis in *Gtl2Δ5'Neo *embryos**. A) Imprinting assay for *Dlk1 *in wild type (left) and *Gtl2Δ5'NeoPat *(right) embryos. B) Imprinting assay for *Dlk1 *in wild type (left), *Gtl2Δ5'NeoMat*^129 ^(center) and *Gtl2Δ5'NeoMat*^*B*6 ^(right) embryos. The *Dlk1 *polymorphism is an "A" in D embryos and a "G" in C embryos; the polymorphic base is boxed on each chromatogram. C) Imprinting assay for *Gtl2 *in *Gtl2Δ5'NeoPat *embryos. D) Imprinting assay for *Gtl2 *in *Gtl2Δ5'NeoMat*^129 ^and *Gtl2Δ5'NeoMat*^*B*6 ^embryos. The *Gtl2 *polymorphism alters an *Sfc*I restriction enzyme site, with the site present in D and absent in C; the D and C bands are indicated at the right.

For imprinting analysis after paternal inheritance, *Gtl2Δ5'Neo *N_2 _males were crossed directly to Cg12 females (see Fig. 9). This is different than the strategy used for *Gtl2lacZPat *mice, and does not generate data for both 129 and B6 backgrounds, but has the advantage that the animals analyzed for imprinting were the same generation (N_3_) as those analyzed for gene expression. For imprinting analysis after maternal inheritance, *Gtl2Δ5'Neo *N_2 _females were crossed to 129 or B6 males, then their female progeny crossed to Cg12 males and sacrificed for embryo recovery. Imprinting analysis showed that *Dlk1 *imprinting is maintained in *Gtl2Δ5'NeoPat *embryos (Fig. 6A). In *Gtl2Δ5'NeoMat*^129 ^embryos, *Dlk1 *levels are 108% of wild type, suggesting the 8% increase in message may arise from a slight relaxation of the maternal *Dlk1 *allele. The sequence-based *Dlk1 *imprinting assay is not quantitative, however, and this small degree of relaxation, if present, was not visible as an "A" peak greater than the background observed in wild type (Fig. 6B). In *Gtl2Δ5'NeoMat*^*B*6 ^embryos, however, where *Dlk1 *expression is 127% of wild type, loss of *Dlk1 *imprinting was variably observed in the sequencing assay (Fig. 6B). *Gtl2 *imprinting was lost in *Gtl2Δ5'NeoPat *embryos (Fig. 6C), but was retained in *Gtl2Δ5'NeoMat*^129 ^and *Gtl2Δ5'NeoMat*^*B*6 ^embryos (Fig. 6D). These results are quite similar to those seen in *Gtl2lacZ*, with overexpression/loss of imprinting of the normally silent gene on the chromosome carrying the integration, and decreased expression of the normally active gene on that chromosome (Table 1). The effects of genetic background are also significant in *Gtl2Δ5'Neo *mice.

### Excision of *Neo *from the *Gtl2Δ5'Neo *allele results in recovery of *Dlk1-Gtl2 *imprinting

Excision of *Neo *from *Gtl2Δ5'Neo *mice was accomplished using a mouse strain that expresses Cre recombinase from the *EIIa *promoter, generating *Gtl2Δ5' *animals (Fig. 4A) [36]. *Gtl2Δ5' *mice were bred as described for *Gtl2Δ5'Neo*, and offspring were analyzed for the expected paternal dwarfism. Surprisingly, animals inheriting the *Gtl2Δ5' *allele paternally (*Gtl2Δ5'Pat*) showed no dwarfism after crosses to either 129 or B6 females (data not shown). When *Gtl2Δ5'Pat *embryos were analyzed for expression and imprinting of *Dlk1*-*Gtl2*, both parameters were found to be wild type (data not shown). Mice inheriting the *Gtl2Δ5' *allele maternally (*Gtl2Δ5'Mat*), also showed no growth phenotype or change in the expression/imprinting of *Dlk1*-*Gtl2 *(data not shown). The dwarfism effect and loss of imprinting seen in the *Gtl2Δ5'Neo *mice therefore does not result from the genomic deletion, but is due to the presence of the *Neo *integration itself. The parallels between the *Gtl2lacZ *and *Gtl2Δ5'Neo *mice are striking. The presence of exogenous sequences upstream of the *Gtl2 *DMR, *lacZ*/β-*actin-Neo *at -1.7 kb in the *Gtl2lacZ *mice, and *Pgk-Neo *at -1.3 kb in the *Gtl2Δ5'Neo *mice, cause loss of imprinting of *Dlk1*-*Gtl2 *upon both paternal and maternal inheritance. These data add further evidence to the growing list of insertional *Neo *effects seen in transgenic and targeted mice, but more importantly, they suggest that insertional mutagenesis upstream of *Gtl2 *perturbs imprinting within the region.

### *Gtl2Δ5'NeoPat *mice show loss of allele-specific methylation at the *Gtl2 *DMR

**Figure 7:**
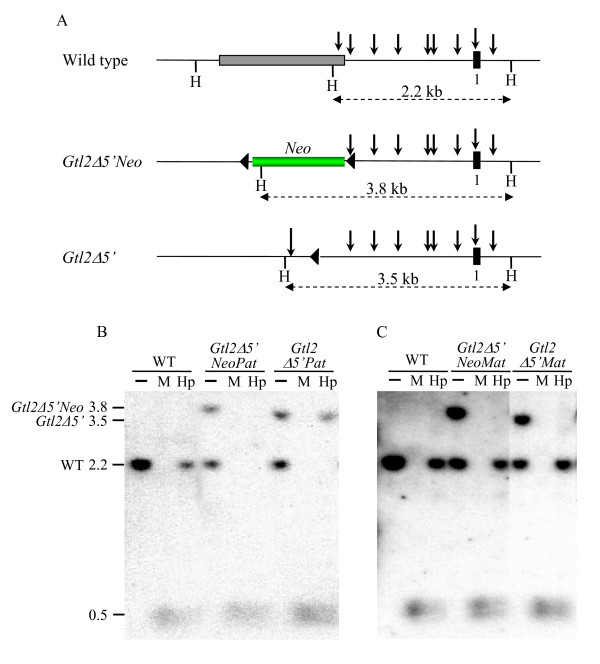
**Methylation analysis of the *Gtl2 *DMR in *Gtl2Δ5'NeoPat *and *Gtl2Δ5'Pat *embryos**. A) Schematic of the *Gtl2 *DMR region in wild type, *Gtl2Δ5'Neo *and *Gtl2Δ5' *alleles. The grey box represents the *Gtl2Δ5' *deletion region, the green box represents the *Neo *gene and the black box represents *Gtl2 *exon 1. The vertical arrows represent recognition sites for the *Msp*I/*Hpa*II restriction enzymes, and the loxP sites are indicated by black triangles. The methylation assay detects a 2.2-kb endogenous *Hinc*II fragment that spans the *Gtl2 *DMR. Modifications in the *Gtl2Δ5'Neo *and *Gtl2Δ5' *alleles generate 3.8-kb and 3.5-kb fragments, respectively. B) Methylation assay for the *Gtl2 *DMR in *Gtl2Δ5'NeoPat*^129 ^and *Gtl2Δ5'Pat*^129 ^embryos. C) Methylation assay for the *Gtl2 *DMR in *Gtl2Δ5'NeoMat*^129 ^and *Gtl2Δ5'Mat*^129 ^embryos. Restriction sites are, H, *Hinc*II, Hp, *Hpa*II and M, *Msp*I.

One mechanism by which the *Gtl2lacZ *and *Gtl2Δ5'Neo *integrations could alter imprinting is if they caused epigenetic changes at the adjacent *Gtl2 *DMR, which is normally methylated on the paternally inherited allele [1,3,28]. The methylation status of the *Gtl2 *DMR was therefore analyzed by Southern blotting in *Gtl2Δ5'Neo *and *Gtl2Δ5' *mice. Genomic DNA from midgestation embryos was digested with the enzyme *Hinc*II in combination with *Msp*I, which cuts both methylated and unmethylated DNA, or *Hpa*II, which cuts only unmethylated DNA (Fig. 7A). While digestion of the wild type allele gives a 2.2-kb *Hinc*II fragment, the *Gtl2Δ5'Neo *and *Gtl2Δ5' *alleles generate fragments of 3.8 kb and 3.5 kb, respectively, allowing allele-specific analysis of *Gtl2 *DMR methylation. Digestion of wild type DNAs with *Hinc*II/*Hpa*II shows that approximately 50% of the signal seen with *Hinc*II alone remains, representing the methylated paternal allele that is not cut by *Hpa*II (Fig. 7B). After *Hinc*II/*Hpa*II digestion of *Gtl2Δ5'NeoPat*^129 ^or *Gtl2Δ5'NeoPat*^*B*6 ^DNA, however, no signal remains for the mutant allele, indicating that the paternal *Gtl2 *DMR methylation is lost in *Gtl2Δ5'NeoPat *embryos (Fig. 7B and data not shown). Importantly, *Neo *excision restored paternal methylation of the *Gtl2 *DMR in *Gtl2Δ5'Pat *embryos. Loss of paternal *Gtl2 *DMR methylation was also seen in G*tl2lacZPat*^129 ^embryos, but not in *Gtl2lacZPat*^*B*6 ^embryos (data not shown), in agreement with the fact that *Gtl2lacZPat*^*B*6 ^mice do not display loss of imprinting. These data show that altered expression of *Dlk1*-*Gtl2 *is associated with loss of paternal methylation at the *Gtl2 *DMR. No change in methylation of the (normally unmethylated) maternal *Gtl2 *DMR was observed in any *Gtl2Δ5'NeoMat *(Fig. 7C) or *Gtl2lacZMat *embryos (data not shown).

### The methylation status of other *Dlk1-Gtl2 *regions is not altered

The promoter of the *Dlk1 *gene is normally unmethylated on both parental alleles [1,28]. The *Dlk1 *DMR located within the 3' region of the *Dlk1 *gene is partially methylated on the paternal allele, but unmethylated on the maternal allele [28]. Since *Dlk1 *expression is decreased in *Gtl2lacZPat*^129 ^mice, changes in methylation at the *Dlk1 *gene itself might be involved in this decrease. Southern analysis of the methylation status of both regions in *Gtl2lacZPat*^129 ^embryos, however, showed them to be indistinguishable from wild type (data not shown). The IG DMR is believed to represent the gametic mark for the *Dlk1*-*Gtl2 *locus, although its functional role appears to be limited to the maternal chromosome [28,29]. This region is normally paternally methylated and maternally unmethylated, and changes in this methylation might alter imprinting in the region. Southern analysis of IG DMR methylation in *Gtl2lacZPat*^129 ^embryos, however, showed no changes from wild type (data not shown). The *Gtl2 *first intron contains a conserved recognition sequence for the CCCTC-binding factor (CTCF), a zinc finger protein shown to be a regulator of imprinting at several loci [2,28,31,37-40]. This region carries largely paternal-specific methylation, and Southern blotting was used to assay for any changes in this pattern in *Gtl2Δ5'Neo *embryos. No differences in methylation of the CTCF-binding site region were observed between wild type and *Gtl2Δ5'Neo *or *Gtl2Δ5' *embryos (data not shown). Analysis of all known DMRs at the *Dlk1*-*Gtl2 *locus, therefore, shows that only changes in the *Gtl2 *DMR correlate with loss of imprinting.

### Expression of the *Gtl2lacZ *and *Gtl2Δ5'Neo *transgenes

Although the *lacZ *gene within the *Gtl2lacZ *transgene lacks its own promoter, the gene is expressed beginning in early embryogenesis, either by read-through from the *β-actin *promoter of the adjacent *Neo *gene, or from a cryptic promoter within the transgene or upstream sequences [27]. This expression is irrespective of parental inheritance, indicating that it is not expression of the transgene alone that confers the different paternal and maternal *Gtl2lacZ *effects. Rather it is likely that expression of the *Gtl2lacZ *transgene interacts with the specific imprinting machinery that controls each allele. RT-PCR analysis of *Gtl2Δ5'NeoPat *and *Gtl2Δ5'NeoMat *embryos showed that the *Neo *gene carried by this allele is also expressed after both maternal and paternal inheritance (data not shown).

### Multiple linked genes are coregulated at the *Dlk1-Gtl2 *locus

Imprinted genes are often subject to coregulation, in which a single regulatory element, or group of elements, controls multiple genes within a domain. At the *Igf2*-*H19 *locus, for example, there are shared regulatory elements for both expression and imprinting [41,42]. At the *Dlk1*-*Gtl2 *locus, the *Callipyge *(*CLPG*) sheep mutation results in overexpression of at least four linked genes in skeletal muscle (*DLK1*, *GTL2*, *PEG11 *and *MEG8*), with no effect on their imprinting [43]. This suggests that the *CLPG *mutation activates an enhancer (or inactivates a repressor) for skeletal muscle expression of these genes. This mutation has been localized to a position between the *Dlk1 *and *Gtl2 *genes, near to, but distinct from, the IG-DMR [44]. The function of the IG-DMR, which regulates maternal chromosome imprinting of multiple genes in the domain, also suggests co-regulation [29].

**Figure 8:**
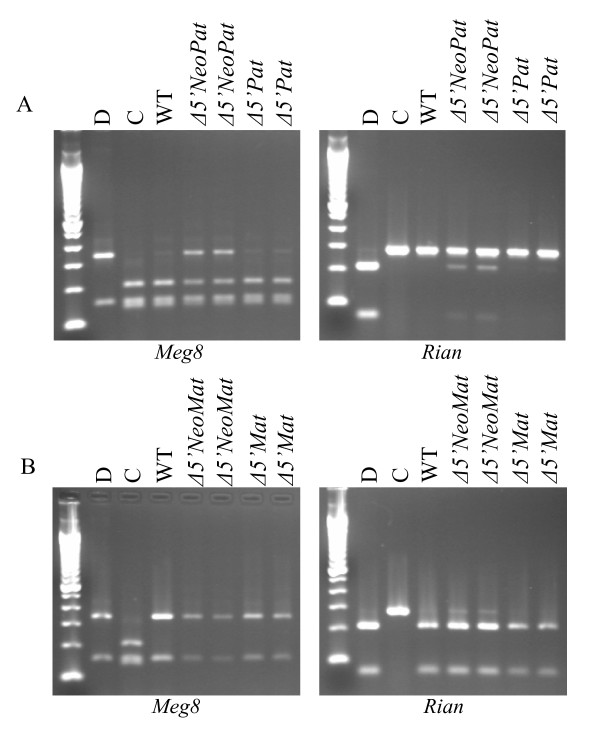
***Meg8*/*Rian *imprinting analysis in *Gtl2Δ5'Neo *embryos**. A) Imprinting assay for the *Meg8 *(left) and *Rian *(right) genes in *Gtl2Δ5'NeoPat*^129 ^and *Gtl2Δ5'Pat*^129 ^embryos. B) Imprinting assay for the *Meg8 *(left) and *Rian *(right) genes in *Gtl2Δ5'NeoMat*^129 ^and *Gtl2Δ5'Mat*^129 ^embryos. The *Meg8 *imprinting assay detects an *Nla*III polymorphism, with the site present in C mice and absent in D mice. The *Rian *imprinting assay detects a *Hinf*I polymorphism, with the site present in D mice, and absent in C mice.

Since the *Gtl2lacZ *and *Gtl2Δ5'Neo *mutations cause a loss of imprinting of both *Dlk1 *and *Gtl2*, it was possible they also deregulate other genes in the cluster. To address this question, imprinting assays were developed for the *Meg8 *and *Rian *genes located distal to *Gtl2 *(Fig. 1A). The *Meg8 *gene is maternally expressed in sheep, and is believed to produce a noncoding RNA [9]. The *Rian *gene is maternally expressed in the mouse, and produces a series of C/D snoRNAs [10,11]. These genes were analyzed separately in this work, but it is likely they represent alternately spliced forms of a single primary transcript (Kalinina & Schmidt, unpublished). The *Meg8 *imprinting assay utilizes an *Nla*III restriction polymorphism, with the site present in C mice and absent in D mice. A second *Nla*III site in the amplified PCR product results in a pattern of three fragments for the C allele, and two fragments for the D allele. The *Rian *imprinting assay detects a *Hinf*I polymorphism, with the site present in D mice and absent in C mice. After crosses to Cg12 mice, *Gtl2Δ5'NeoPat*^129 ^embryos showed biallelic expression of both *Meg8 *and *Rian*, indicating activation of the normally silent paternal alleles of these genes (Fig. 8A). *Gtl2Δ5'NeoMat*^129 ^embryos did not show loss of imprinting of either *Meg8 *or *Rian *(Fig. 8B). Attempts to analyze the imprinting of the *Rtl1*/*αRtl1 *genes in *Gtl2Δ5'Neo *mice have so far proven impossible, as there are no sequence polymorphisms in these genes between D and C mice.

## Discussion

### Exogenous transcription units in the *Gtl2 *upstream region alter *Dlk1-Gtl2 *imprinting

The data presented here document the molecular characterization of two mouse lines with insertional mutations upstream of the *Gtl2 *gene. *Gtl2lacZ *mice carry a 15-kb insertion containing multiple copies of a promoterless-*lacZ*/*β-actin*-*Neo *gene trap vector. *Gtl2Δ5'Neo *animals have a 2.8-kb endogenous deletion that is replaced by a 1.9-kb *Pgk*-*Neo *cassette. Both integrations contain bacterial DNA including the *Neo *gene, and both *Neo *genes are under the control of constitutive promoters. There are key differences in the two integrations, however, including the size of the insertion, the specific promoters used, and the deletion of endogenous sequence. The integrated reporter genes are both transcribed but in opposite directions, with the *Gtl2lacZ *transgene transcribed towards *Dlk1*, and the *Gtl2Δ5'Neo *transgene transcribed towards *Gtl2*. Despite these differences, *Gtl2lacZ *and *Gtl2Δ5'Neo *mice show similar phenotypes. Both mutations cause partial neonatal lethality upon paternal inheritance, with dwarfism of surviving mice, and changes in expression and imprinting of linked genes after paternal and maternal inheritance. The mechanism of action is therefore likely to be the same in both lines. The integrations themselves are causative for these effects, rather than deletion of endogenous sequence, since excision of the *Neo *cassette from the *Gtl2Δ5'Neo *allele restores a normal phenotype.

While this work was in progress, another group also reported data on the loss of imprinting in *Gtl2lacZ *mice [45]. The results from this work are largely consistent with those presented here, but there are some differences as well. Sekita et al also observe a 50% reduction in *Dlk1 *RNA levels after paternal inheritance of the *Gtl2lacZ *mutation, and show that *Dlk1 *imprinting is maintained in these mice. The data presented here, however, report a concomitant increase in the levels of *Gtl2 *RNA to 127% of wild-type, with biallelic expression, while Sekita reports no change in *Gtl2 *levels. These authors do see biallelic expression of *Gtl2*, however, indicating that there is activation of the paternal *Gtl2 *allele in their mice, but perhaps not to a level measurable by the assays used. After maternal *Gtl2lacZ *inheritance, the data presented here show decreased *Gtl2 *RNA levels to 50–60% of wild type with no loss of imprinting, and an increase in *Dlk1 *RNA to ~130% of wild type with biallelic expression. Sekita et al observe levels of *Gtl2 *decreased to 20–40% of wild type with no loss of imprinting, but report no changes in *Dlk1 *expression or imprinting. Both groups report a complete loss of DNA methylation on the paternal *Gtl2 *DMR after paternal inheritance of the *Gtl2lacZ *mutation. While the basis for these largely quantitative differences is not known, it is likely that different genetic backgrounds, and/or different assay techniques are responsible.

### Loss of *Gtl2 *DMR methylation may underlie loss of imprinting in *Gtl2lacZPat *and *Gtl2Δ5'NeoPat *mice

The biallelic expression of *Gtl2 *observed in *Gtl2lacZPat *and *Gtl2Δ5'NeoPat *animals may result from loss of paternal-specific methylation at the *Gtl2 *DMR. Methylation is a key regulator of genomic imprinting, and the position of the *Gtl2 *DMR suggests it could directly silence the paternal *Gtl2 *allele. Other work from our laboratory has demonstrated that the loss of *Gtl2 *DMR methylation in *Gtl2Δ5'NeoPat *mice is also correlated with increased histone acetylation at the paternal *Gtl2 *DMR (Carr et al, submitted). While additional changes to unknown regulatory regions cannot be ruled out, all other *Dlk1*-*Gtl2 *DMRs are unaltered with regard to methylation and chromatin structure. The maternally expressed *Meg8*/*Rian *genes lie more than 70 kb from the *Gtl2 *DMR, suggesting a more complicated mechanism than direct silencing would need to exist, perhaps a long-range, methylation-dependent silencer within the *Gtl2 *DMR. It should be noted, however, that since the methylation of the *Gtl2 *DMR occurs post-fertilization, this modification may not be involved in initiating the silencing of the paternal *Gtl2 *allele, but only in maintenance of the silent state. Additionally, it is possible that the *Gtl2 *integration prevents paternal *Gtl2 *silencing by a mechanism unrelated to the loss of DMR methylation, with the lack of methylation being secondary to continued expression of the paternal *Gtl2 *allele.

Hypermethylation of integrated transgenes in the mouse is common, and this methylation frequently spreads into surrounding endogenous sequences [46]. The causative sequences are usually bacterial or viral, and are targeted by the cellular methylation machinery because of their resemblance to endogenous retroviral elements, and/or their tendency to integrate in repetitive arrays. The *Gtl2lacZ *and *Gtl2Δ5'Neo *integrations, however, prevent the methylation of a normally methylated endogenous sequence. Possible mechanisms for this effect are discussed below.

### Parental- and strain-specific effects modify the *Gtl2lacZ *and *Gtl2Δ5'Neo *phenotypes

The *Gtl2lacZ *and *Gtl2Δ5'Neo *mutations are modified by genetic background, with *Gtl2lacZPat *exhibiting a phenotype only in crosses to 129 females, while the *Gtl2Δ5'NeoPat *mutation shows an effect in both 129 and B6 crosses. The changes in gene expression and imprinting in *Gtl2lacZPat *and *Gtl2Δ5'NeoPat *mice correlate with altered methylation at the *Gtl2 *DMR, and may therefore be due to strain-specific modifiers of DMR methylation. Parental- and strain-specific differences in transgene methylation are well documented [47-50]. A troponin transgene, for example, is methylated in the mouse when passed maternally, but is fully or partially unmethylated when passed paternally [51]. This variation in methylation is largely dependent on the genetic background of the non-transgenic female that is crossed to the transgenic male. This is reminiscent of the ability of a single cross to a 129 or B6 female to modify the effects of the *Gtl2lacZPat *and *Gtl2Δ5'NeoPat *mutations. In general, the B6 strain has been shown to be more highly methylating than are 129 strains. The normal phenotype of *Gtl2lacZPat*^*B*6 ^mice might therefore result from a greater ability of this high-methylating background to accomplish *Gtl2 *DMR methylation despite the presence of the integration. The fact that the *Gtl2Δ5'NeoPat*^*B*6 ^mutation produces a phenotype, however, indicates that these strain-dependent modifiers affect the two integrations differently.

### Models for the *Gtl2lacZPat *and *Gtl2Δ5'NeoPat *effects on the *Dlk1-Gtl2 *region

The question remains how the presence of the *Gtl2lacZ *and *Gtl2Δ5'Neo *integrations prevent paternal methylation of the *Gtl2 *DMR. Several possibilities must be considered. First, it is possible the integrations physically disrupt a regulatory element that directs methylation. However, the observation that a deletion of this region has no effect (*Gtl2Δ5'*) suggests that this is not the case. Second, the integrations may have altered the relative positioning of an upstream element that controls *Gtl2 *DMR methylation. The 15-kb *Gtl2lacZ *integration might move a *Gtl2 *methylating element too far away from the *Gtl2 *DMR for proper function. The deletion associated with the *Gtl2Δ5'Neo *mutation, however, brings the *Gtl2 *upstream sequence 0.9 kb closer to the *Gtl2 *DMR than in wild type animals. A third possibility is that transcriptional interference from the expressed *lacZ/β-actin-Neo *and *Pgk1-Neo *genes might interfere with the ability of DNA methyltransferase to establish a silent paternal chromatin structure within this region. Transcription from the *Gtl2Δ5'Neo *integration through the *Gtl2 *DMR might prevent the binding of factors that initiate or maintain methylation of the paternal *Gtl2 *DMR. This argument is complicated by the fact that the *Gtl2lacZ *transgene is transcribed away from the *Gtl2 *DMR, but recent work has shown that transcriptional interference can occur between adjacent promoters organized in all possible directional combinations [52]. Further studies of the *Gtl2 *DMR and upstream region will be necessary to define the requirements for proper methylation.

Interestingly, a genomic element has been identified that can protect both a transgene, and adjacent endogenous sequences, from genomic methylation [53]. This IE (island element) was identified within the *Aprt *gene, but can also protect heterologous sequences within the context of an integrated mouse transgene. While the mechanism of IE function is not known, this element appears to function during the wave of *de novo *methylation that occurs at implantation, as IE deletion beyond this point has no effect. One possible explanation for the loss of *Gtl2 *DMR methylation would be a similar type of element present within the *Gtl2lacZ *and *Gtl2Δ5'Neo *transgenes.

### Models for the *Gtl2lacZMat *and *Gtl2Δ5'NeoMat *effects on the *Dlk1-Gtl2 *region

Biallelic *Dlk1 *expression in the *Gtl2lacZMat *and *Gtl2Δ5'NeoMat *animals suggests that these animal models may also provide information about the mechanism by which the maternal *Dlk1 *allele is silenced. A number of possibilities can be suggested for why *Dlk1 *imprinting is lost, and these models may function alone or in combination. First, a model has been proposed in which the maternally expressed noncoding RNAs at the *Dlk1*-*Gtl2 *locus act to silence *Dlk1 *posttranscriptionally [54,55]. If this model is proven to be true, the decreased levels of *Gtl2*, and presumably other maternal noncoding RNAs, found in the *Gtl2lacZMat *and *Gtl2Δ5'NeoMat *mice might allow for increased levels of *Dlk1*. The model as proposed allows only for regulation in *trans*, however, while the *Gtl2lacZ *and *Gtl2Δ5'Neo *model systems show activation of *Dlk1 *in *cis *to the mutation.

Second, it is possible that the *Gtl2 *DMR itself has a role in silencing the maternal *Dlk1 *allele. The maternal *Gtl2 *DMR is not normally methylated, and does not acquire methylation in the *Gtl2lacZMat *and *Gtl2Δ5'NeoMat *animals, so it would have to function by another mechanism. One manner in which this region might promote *Gtl2 *transcription, while silencing *Dlk1*, would be if it carried a strong enhancer that preferentially interacted with the *Gtl2 *promoter. Methylation of the DMR would inactivate this enhancer paternally, and the *Gtl2lacZ *and *Gtl2Δ5'Neo *integrations might alter its structure. Similarly, the region might contain a long-range repressor specific for the *Dlk1 *gene. Lastly, the integrations might act by altering chromatin structure over a larger genomic region, disrupting a chromatin conformation that favors *Gtl2 *expression and *Dlk1 *silencing.

Activation of the normally silent allele of *Dlk1 *or *Gtl2*, on a given chromosome, results in decreased expression of the normally active gene on that chromosome. This is reminiscent of effects seen at *Igf2*-*H19*, where activation of both genes on a single chromosome leads to expression at levels less than wild type, likely due to competition for shared enhancers [42]. The locations of the enhancers that regulate tissue-specific *Dlk1*-*Gtl2 *expression are largely unknown, but the genes are coexpressed in many neuroendocrine cell types. The reciprocal changes in gene expression seen after loss of *Dlk1*-*Gtl2 *imprinting suggest that the *Dlk1 *and *Gtl2 *genes may share common enhancers for those tissues in which they are coexpressed.

### Decreased *Dlk1 *expression may be responsible for the dwarfism of the *Gtl2lacZPat *and *Gtl2Δ5'NeoPat *mice

The neonatal lethality and dwarfism of the *Gtl2lacZPat *and *Gtl2Δ5'NeoPat *mice are similar to mice carrying a paternal deletion of the *Dlk1 *gene, although *Dlk1 *null mice also show skeletal, adipose and eye defects [25]. These data suggest that it is likely to be the decreased level of *Dlk1 *in the *Gtl2lacZPat *and *Gtl2Δ5'NeoPat *animals that is the cause of their lethality and dwarfism. The proportionate dwarfism of the *Gtl2lacZPat*, *Gtl2Δ5'NeoPat *and *Dlk1 *null mice is reminiscent of animals with reduced levels of growth hormone, and there is evidence for interactions between the *Dlk1 *and GH pathways [56-59]. *Gtl2lacZPat *and *Gtl2Δ5'NeoPat *mice may not display the other effects seen in *Dlk1 *null mice because *Dlk1 *in these animals is only decreased, not absent, and growth may be the most sensitive indicator of *Dlk1 *levels. It is notable that those animals which show the most severe phenotype, *Gtl2Δ5'NeoPat*^*B*6^, also show the lowest levels of *Dlk1*. The *Gtl2lacZPat *and *Gtl2Δ5'NeoPat *animals also show loss of imprinting of *Gtl2 *and *Meg8*/*Rian*, and it cannot be ruled out that changes in the levels of these transcripts are causative for the dwarfism. Neither of these transcripts produces a protein, however, and they have not been shown to have any physiological role. If *Dlk1 *functions as a growth factor, it might be expected that overexpression of *Dlk1 *would confer an overgrowth phenotype. *Gtl2Δ5'NeoMat*^129 ^mice show levels of *Dlk1 *up to 131% of wild type, yet no overgrowth is detected in these animals. The generation of transgenic animals expressing greater levels of *Dlk1 *will be required to determine the effects of overexpression of this gene.

## Conclusion

The work presented here demonstrates that the integration of exogenous DNA sequences upstream of the *Gtl2 *DMR, at -1.7 kb in the *Gtl2lacZ *mutation and -1.3 kb in the *Gtl2Δ5'Neo *mutation, causes loss of imprinting and altered expression of *Dlk1 *and *Gtl2*. Mice inheriting either mutation paternally show partial neonatal lethality and dwarfism of surviving animals, a phenotype that may be due to the decreased levels of *Dlk1 *found in these mice. Both models also show a loss of the paternal-specific methylation at the *Gtl2 *DMR in *cis *to the mutation. Animals in which the *Gtl2Δ5'Neo *selectable marker is removed using Cre recombinase are indistinguishable from wild type, indicating that it is the presence of the integrated DNA causing the mutant phenotypes. These studies present further evidence for the coregulation of the *Dlk1 *and *Gtl2 *genes, and support a role for *Dlk1 *as an important neonatal growth factor. The loss of *Gtl2 *DMR methylation is particularly intriguing; in future experiments it will be important to define what role this region may play in imprinting regulation.

## Methods

### *Gtl2lacZ *mice

The *Gtl2lacZ *mice were obtained on a B6 × 129 × BALB/c background, and were maintained on a B6 × 129 background by alternating breeding partners at each generation. *Gtl2lacZ *mice were genotyped by PCR using primers that detect the full-length *lacZ*-*Neo *integration, as well as a spontaneously occurring internal deletion [27]. Although the phenotype of animals with the full and deleted integrations display similar phenotypes, only mice with the full insertion were analyzed. The genotyping primers were: Gtl2lacZ3, 5'-CAACTTAATCGCCTTGCAGC-3' with Gtl2lacZ4, 5'-CCAGATAACTGCCGTCACTCC-3', which detect the full transgene; and Gtl2trlacZ, 5'-AGCCACAGACGTCATTATGC-3' with Gtl2lacZ4, which detect the deleted integration. PCR conditions were 94°C, 1 min, 62°C, 1 min, 72°C, 1 min, for 35 cycles. Full details of all breeding strategies for the mice analyzed in this work are given in Figure 9.

### *Gtl2Δ5'Neo *mice

The *Gtl2Δ5'Neo *targeting construct was generated using the vector ploxPNT, which carries a Neomycin resistance gene flanked by loxP sites. Genomic DNA was isolated from the RPCI-22 BAC clone 28G5. The 5' arm was a 1.4-kb *Eco*RI-*Bgl*II fragment spanning the region from -5516 to -4094 bp relative to the *Gtl2 *transcriptional initiation site, and the 3' arm was a 6.0-kb *Nae*I-*Eco*RI fragment spanning the region from -1348 to +4608 bp. The targeting construct was linearized and transfected into E14 embryonic stem (ES) cells using a Bio-Rad GenePulser at 250 V/500 μF. Transfected ES cells were subjected to selection in G418, and clones picked to 96 well plates for analysis [60]. ES cell DNAs were analyzed by Southern blotting using either *Bst*EII or *Kpn*I digests, and hybridized with a PCR generated probe corresponding to nucleotides -5401 to -6394 relative to *Gtl2 *(primers OL306, 5'-ACTCTTCTCTCTCCAATGGCAAG-3', and OL307, 5'-CATTCAACTGCCTACAGTTAGGGAG-3'). The *Bst*EII digest detects a 6.6-kb band in wild type ES cells and a 5.7-kb band in properly targeted ES cells; the *Kpn*I digest detects 4.2-kb wild type and 7.2-kb targeted bands. Cells from correctly targeted ES clones were injected into B6 blastocysts by the UIC Transgenic Production Service, and the blastocysts implanted in pseudopregnant ICR females. Resulting chimeras were bred to B6 mice to assay for germline transmission of the targeted allele, and N_1 _animals crossed to B6 to give N_2 _mice. During maintenance and analysis, *Gtl2Δ5'Neo *mice were genotyped using a forward primer upstream of the deletion, and a reverse primer in the *Neo *gene. The forward primer was OL521, 5'-GCGATTACCCTTGGGTTACTGC-3' and the reverse primer was OL708, 5'-AACTTCTGACTAGGGGAGGAG-3'. PCR conditions were 95°C, 1 min, 57°C, 40 sec and 72°C, 40 sec, for 35 cycles. The *Neo *cassette was removed by crossing *Gtl2Δ5'Neo *males to *EIIa-Cre *females (Jackson Labs, B6.FVB-Tg(EIIa-Cre)C5379 Lmgd/J) [36]. *EIIa-Cre *mice often give mosaic excision in the F_1 _offspring, a result of Cre activation after the first cleavage division. DNA was isolated from tail biopsies of progeny and genotyped using the OL521/OL708 PCR assay described above, and also with primers OL521 and OL285, 5'-TAGGATGCCACTGTGACTCGG-3', which detect the excised allele. PCR conditions were 95°C, 1 min, 59°C, 40 sec and 72°C, 40 sec, for 35 cycles. Mice showing only the excised allele by tail DNA PCR were verified by a test breeding to ensure they passed only the *Gtl2Δ5' *allele.

### Southern blotting and methylation analysis

Genomic DNA was extracted using proteinase K digestion and phenol:chloroform extraction. For Southern blotting, 10 μg of DNA was digested with the appropriate restriction enzyme(s), separated on 1× TAE agarose gels, and transferred to Hybond N+ membranes (GE Healthcare). Membranes were incubated in hybridization buffer (5× SSPE, 0.5% SDS, 5× Denhardt's solution, 50 ng/ml salmon sperm DNA) overnight at 65°C. Washing conditions were: twice in 2× SSPE, 0.1% SDS, once in 1× SSPE, 0.1% SDS, and once in 0.1× SSPE, 0.1% SDS, all at 65°C. Membranes were exposed to X-ray film at -80°C with an intensifying screen. For methylation analysis of the *Gtl2 *DMR, DNA was digested with *Hinc*II in combination with *Msp*I or *Hpa*II, and hybridized with a 532-bp *Sac*I-*Xho*I fragment spanning nucleotides +19 to +551 of the *Gtl2 *gene.

### Northern blotting

Total RNA was extracted from embryos using TRIzol reagent (Invitrogen). Ten micrograms of total RNA was separated on 1% formaldehyde-MES agarose gels, blotted to Hybond N+ membranes (GE Healthcare) and hybridized with Express-Hyb solution (Clontech) for 1 hr at 65°C. Membranes were hybridized with the following probes: *Dlk1*, a 735-bp PCR-generated fragment corresponding to nucleotides 685 to 1420 of the *Dlk1 *transcript; *Gtl2*, a 368-bp PCR-generated fragment spanning nucleotides 1482 to 1850 of the *Gtl2 *transcript; and a 1.2-kb fragment of the mouse β-*actin *gene. Membrane washes were: three times in 2× SSC, 0.05% SDS, and twice in 0.1× SSC, 0.1% SDS, all at 50°C. Signal intensity was quantified by Phosphor imaging, and *Dlk1 *and *Gtl2 *levels were normalized to the expression of β-*actin*. *Gtl2lacZ *and *Gtl2Δ5'Neo *animals were analyzed from e12.5 to e17.5; temporal variation in gene expression levels were seen across this time period, particularly for *β-actin*, but relative changes in expression between wild type and mutant animals did not vary with gestational age.

### Imprinting assays

Allele-specific expression of the *Dlk1 *and *Gtl2 *genes was analyzed using previously described single nucleotide polymorphisms between the *M. m. domesticus *(D) allele carried by 129 and B6 mice, and the *M. m. castaneus *(C) allele carried by the Cg12 congenic line [1]. For RT-PCR analysis, 2 μg of total RNA was reverse transcribed using Superscript III (Invitrogen), RT reactions were diluted 1:10, and 2 μl was used for PCR amplification. In all imprinting analyses, control reactions in the absence of reverse transcriptase were negative. *Dlk1 *imprinting analysis involves RT-PCR followed by direct sequencing of a 288-bp fragment of the 3' region of the *Dlk1 *transcript. This assay detects an A/G polymorphism, with the A allele present in D mice and the G allele present in C mice. *Gtl2 *imprinting analysis involves RT-PCR followed by digestion with the restriction enzyme *Sfc*I, with the site present in D mice and absent in C mice.

The *Meg8 *imprinting assay examines a polymorphic *Nla*III restriction site using primers designed against the mouse genomic sequence homologous to that reported for ovine *Meg8 *[9]. Primers were OL552, 5'-TAAGTAATTGCTGAGTGCCTTG-3' and OL553, 5'-TCAGTTGAGCTGGATCACATTA-3'. PCR conditions were 94°C, 30 sec, 55°C, 30 sec and 72°C, 1 min for 33 cycles. The resulting product is 495 bp, and *Nla*III digestion of the D allele generates two fragments of 151 and 344 bp each, while the C allele yields three fragments of 151 bp, 207 bp, and 137 bp. The *Rian *imprinting assay detects a polymorphic *Hinf*I restriction site using primers designed against the GenBank sequence AK017440. Primers were OL596, 5'-GACTCATAGTTCTTTGTCTGGG-3' and OL595, 5'-GTGAAAGGCTGAAGGAGCTG-3'. PCR conditions were 94°C, 30 sec, 56°C, 30 sec and 72°C, 45 sec, for 29 cycles. The resulting product is 281 bp long, and *Hinf*I cuts the B6 allele, yielding fragments of 68 bp and 213 bp.

## Authors' contributions

ES performed the breeding and analysis of the *Gtl2lacZ*, *Gtl2Δ5'Neo *and *Gtl2Δ5' *mice, and assisted with the writing of the manuscript; MC and OA assisted with the breeding and analysis of the *Gtl2Δ5'Neo *mice; EG performed the imprinting analysis of *Meg8 *and *Rian*; AY assisted in the DNA methylation analysis; JS conceived of the project, designed the experiments, performed the *Gtl2Δ5' *targeting and wrote the manuscript. All authors approved the final version of the manuscript.
